# Structural inference embedded adversarial networks for scene parsing

**DOI:** 10.1371/journal.pone.0195114

**Published:** 2018-04-12

**Authors:** ZeYu Wang, YanXia Wu, ShuHui Bu, PengCheng Han, GuoYin Zhang

**Affiliations:** 1 College of Computer Science and Technology, Harbin Engineering University, Harbin, HeiLongJiang, China; 2 School of Aeronautics, Northwestern Polytechnical University, Xi’an, ShaanXi, China; 3 Shaanxi Key Laboratory of Integrated and Intelligent Navigation, Xi’an, ShaanXi, China; University of Virginia, UNITED STATES

## Abstract

Explicit structural inference is one key point to improve the accuracy of scene parsing. Meanwhile, adversarial training method is able to reinforce spatial contiguity in output segmentations. To take both advantages of the structural learning and adversarial training simultaneously, we propose a novel deep learning network architecture called Structural Inference Embedded Adversarial Networks (SIEANs) for pixel-wise scene labeling. The generator of our SIEANs, a novel designed scene parsing network, makes full use of convolutional neural networks and long short-term memory networks to learn the global contextual information of objects in four different directions from RGB-(D) images, which is able to describe the (three-dimensional) spatial distributions of objects in a more comprehensive and accurate way. To further improve the performance, we explore the adversarial training method to optimize the generator along with a discriminator, which can not only detect and correct higher-order inconsistencies between the predicted segmentations and corresponding ground truths, but also exploit full advantages of the generator by fine-tuning its parameters so as to obtain higher consistencies. The experimental results demonstrate that our proposed SIEANs is able to achieve a better performance on PASCAL VOC 2012, SIFT FLOW, PASCAL Person-Part, Cityscapes, Stanford Background, NYUDv2, and SUN-RGBD datasets compared to the most of state-of-the-art methods.

## 1 Introduction

Scene parsing, one of the most fundamental tasks in computer vision, aims at predicting a class label for every pixel of input images, which can be beneficial to a wide scope of intelligent applications, including image-to-caption generation [1], robot task planning [2], action recognition [3], self-driving cars [4], and automatic photo adjustment [5]. A real scene always contains multiple categories of objects, and the appearances of objects are diverse. Because of this, scene parsing belongs to a challenging pixel-level multi-label classification task, which not only cares about the visual appearances of objects, but also takes into account the spatial dependencies among objects. As a consequence, there are two key issues affecting the accuracy of scene parsing in the latest researches: (1) How to extract the effective representations from input images, which contain the precise visual appearance information of objects [6–8]; (2) How to capture the global scene layouts of input images, which encapsulate the explicit global contextual information used to encode the spatial dependencies among objects [9, 10].

Over past years, Convolutional Neural Networks (CNNs) [6–8], one type of deep learning methods, have achieved a big breakthrough on scene parsing. Among these CNNs-based methods [11–21], Fully Convolutional Networks (FCNs) [11], trained to perform dense pixel-wise scene labeling in an end-to-end fashion, takes input images of arbitrary size and produces correspondingly-sized output segmentations with efficient inference and learning. However, the repeated combination of max-pooling and downsampling performed at every layer of the standard CNNs significantly reduces the resolution of feature maps, and leads to the coarse semantic segmentations with non-smooth boundaries. To remedy this issue, DeepLab [12] employs atrous convolutions, also known as dilated convolutions [9], to extract the dense high-resolution features, which increase the chance of obtaining the fine segmentations with smooth outlines. Despite of this, current CNNs-based methods can only capture the contextual information among local areas due to the restricted field-of-view of convolutional operations, which leads to suboptimal performance. To improve the accuracy of scene parsing, the global contextual information of the whole images need to be captured through constructing structural inference model.

To overcome the issue from the pure CNNs-based methods, probabilistic graphical models, such as Markov Random Fields (MRFs) [22, 23] or Conditional Random Fields (CRFs) [24–29], have been adopted to increase the accuracy of scene parsing in a post-processing step. DeepLab-CRF [25] utilizes the bilinear interpolations to upsample the coarse score maps into the same size of input images and applys the fully connected CRFs model to refine the boundaries of segmentations. To optimize the CNNs and CRFs jointly, CRF-RNN [26] formulates conditional random fields as recurrent neural networks and integrates this functional module as one part of CNNs, then trains the whole network end-to-end with the usual back-propagation algorithm. In order to achieve more precise structural inference for scene parsing, DSMs [29] formulates conditional random fields with CNNs-based pairwise potential functions to capture the semantic correlations between neighboring regions, and exploits an efficient piecewise training method to learn the deep structured network jointly. To summarize, these CRFs-based methods usually optimize the classification probabilities by minimizing the CRFs energy function according to the color contrast information. Nevertheless, most CRFs-based methods do not explicitly strengthen the intermediate features extracted from CNNs by incorporating the global contextual information of objects, which leads to suboptimal scene labeling results under complex scenes.

For this purpose, an alternative method focuses on employing long short-term memory networks (LSTMs) [30] with gate and memory structures to capture the global contextual information of objects [31–38], which can be well memorized by sequentially running the LSTMs over all pixels of input images. ReNet [32] designs a novel cascaded model based on pure uni-dimensional LSTMs to obtain a global perspective of input images by performing bidirectional propagation of local contextual information along the vertical and horizontal directions. Moreover, LG-LSTM [33] achieves a novel designed local-global long short-term memory architecture to incorporate the short-range and long-range spatial dependencies among objects into the feature learning over all pixels, and exploits local contextual information from neighboring positions (8 adjacent pixels) and global contextual information from whole images to enhance the visual features learnt from CNNs. Furthermore, Graph-LSTM [34] is the generalization of LG-LSTM [33] from sequential data or multi-dimensional data to general graph-structured data, and constructs an adaptive graph topology to propagate contextual information between adjacent superpixels. In order to enhance the capacity of involving various spatial layouts, PDNs [35] achieves a wider range of contextual information diffusion by stacking multiple LSTMs, and incorporate multi-level contextual information into the procedure of feature learning. With the help of LSTMs, global contextual information can be inferred so as to get a better performance.

Recently, Generative Adversarial Networks (GANs) [39–43] have received much attention in computer vision. The basic idea behind GANs is to train a discriminator in company with a generator. The generator and discriminator are like two competitors playing a minmax game with each other. In this game, the goal of the discriminator is to make every effort to distinguish the fake data (generated by the generator) from the real data (from true data distribution), and the goal of the generator is to try its best to learn a model distribution matching the true data. Based on this idea, GANs have been adopted for a wide scope of tasks, such as super-resolution [44], in-painting [45], and style transfer [46]. In particular, DANs [41] proposes an adversarial training method to optimize a convolutional scene parsing network (generator) along with a discriminator, which encourages the generator to produce the segmentations much closer to the corresponding ground truths. Furthermore, the adversarial training method is able to enforce higher-order consistencies between the predicted segmentations and ground truths so as to reinforce spatial contiguity in the output segmentations.

In this paper, on the basis of our previous IEDNs [47], we propose a deep network architecture called Structural Inference Embedded Adversarial Networks (SIEANs) for pixel-wise scene parsing, which takes both advantages of the spatial structure inference and adversarial training method simultaneously. The generator of the SIEANs, composed of three types of layers, is a novel designed scene parsing network, which has the ability of spatial structure inference to explicitly capture the global contextual information of objects by optimizing the CNNs and LSTMs jointly. Meanwhile, we explore the adversarial training method by combining the adversarial loss function with the standard segmentation loss function, and optimize the generator along with the discriminator. By this way, not only higher-order inconsistencies between the predicted segmentations and corresponding ground truths can be detected and corrected, but also the advantages of each layer in the generator can be exploited fully by refining its parameters. In brief, our SIEANs mainly comprises of the following two adversarial modules:

1. The generator, composed of the feature learning layer (convolutional neural networks), the structural learning layer (long short-term memory networks), and the feature fusion layer (multiple convolutional layers with softmax function), performs pixel-wise scene labeling in an end-to-end fashion, the goal of which is to try its best to generate the segmentations that are able to cheat the discriminator.
  - In the feature learning layer, we take advantages of each layer of CNNs to efficiently extract the hierarchical visual features (HVFs) from RGB images, which consist of different scales of pyramid version representations. Through this way, for each pixel of input images, it is represented by a feature vector which contains the hierarchical visual appearance information of objects.
  - In the structural learning layer, we utilize four uni-dimensional LSTMs as a whole to sweep over the HVFs pixel by pixel along four different directions, and infer the pixel-wise spatial structure features (SSFs), which comprise of the explicit global contextual information used to encode the spatial dependencies among objects.
  - In the feature fusion layer, we exploit multiple convolutional layers to fuse the HVFs and SSFs into the hybrid features which encapsulate the comprehensive semantic information of objects, then utilize softmax function to perform pixel-wise scene labeling according to the fused hybrid features.
2. The discriminator, with an architecture of CNNs, competes with the generator via an adversarial training method, the goal of which is to make every effort to distinguish the predicted segmentations from corresponding ground truths.

As a consequence, compared to the previous methods, the contributions of our SIEANs are as follows:

1. Explicit spatial structure learning: For the reason that the receptive fields of upper convolutional layers are large, we also use the feature maps extracted from upper layers to implicitly learn the local contextual information from neighboring positions. In addition, we design a novel structural learning layer to explicitly infer the global contextual information of objects in four different directions, which is able to describe the spatial distributions of objects in a more comprehensive and accurate way. Through this way, not only the visual appearance information of objects is used to estimate their classes, but also the local and global contextual information among objects is utilized to optimize classification and avoid misclassification. Thereby the performance of the SIEANs can be better.
2. Multi-modal features fusion: We set up a feature fusion layer to learn the HVFs and SSFs together, and explore the comprehensive non-linear relationships between them. By this way, the precise visual appearance information and explicit global contextual information can be encapsulated together to improve the performance of the SIEANs.
3. Effective adversarial training: We optimize our scene parsing network (generator) via an adversarial training method, which has a high capacity to detect and correct mismatches in a wide range of higher-order statistics between the predicted segmentations and corresponding ground truths, and is flexible enough to refine the parameters of the generator to exploit full advantages of our scene parsing network.

We evaluate our proposed SIEANs on a number of popular scene parsing datasets, including PASCAL VOC 2012 [48], SIFT FLOW [49], PASCAL Person-Part [50], Cityscapes [51], Stanford Background [52], NYUDv2 [53], and SUN-RGBD [54]. Compared to the previous state-of-the-art methods, our SIEANs achieves a better performance on scene parsing.

## 2 The proposed methods

**Fig 1:**
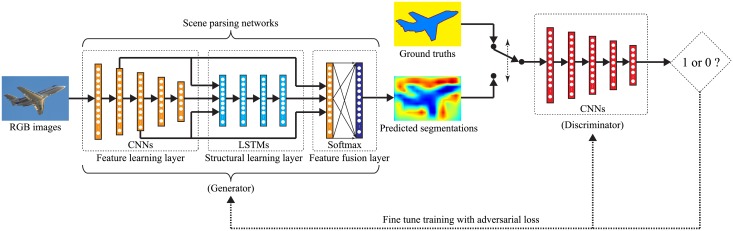
The architecture of our proposed SIEANs. The SIEANs mainly comprises of two adversarial modules: the generator and discriminator. The generator, composed of the feature learning layer (convolutional neural networks), the structural learning layer (long short-term memory networks), and the feature fusion layer (multiple convolutional layers with softmax function), performs pixel-wise scene labeling in an end-to-end fashion. Meanwhile, the discriminator (convolutional neural networks) competes with the generator via an adversarial training method.

The architecture of our proposed SIEANs is based on two adversarial modules: the generator and discriminator. The generator, a novel designed deep network for scene parsing, aims at producing the segmentations that are able to cheat the discriminator. Meanwhile, the discriminator aims at distinguishing the predicted segmentations from the corresponding ground truths. They are like two competitors playing a minmax game with each other. The architecture of the SIEANs is depicted in Fig 1.

### 2.1 Generator

The structure of the generator is mainly composed of three type layers: the feature learning layer, the structural learning layer, and the feature fusion layer. The feature learning layer takes full advantages of each layer of CNNs to extract the hierarchical visual features (HVFs) from RGB images. The structural learning layer, composed of four uni-dimensional LSTMs as a whole, sweeps over the HVFs pixel by pixel along four different directions to infer the spatial structure features (SSFs). The feature fusion layer is set up to learn above two types of features HVFs and SSFs together. And above three layers constitute our scene parsing network, which performs pixel-wise scene labeling in an end-to-end fashion. The details of each layer are introduced as follows.

#### 2.1.1 Feature learning layer

**Fig 2:**
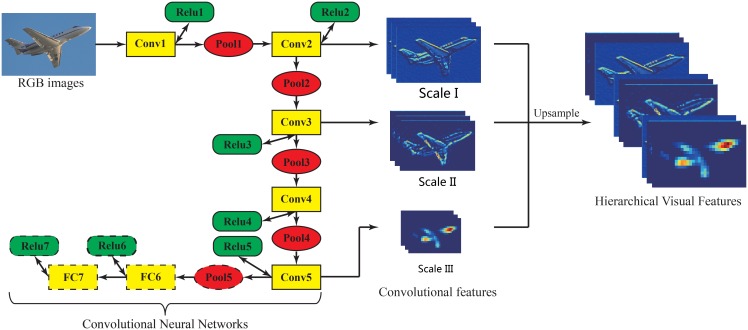
The principle of the feature learning layer. We utilize the modified Deeplab networks to extract the convolutional features from RGB images, and select the features from the 2-th, 3-th, and 5-th layer of the Deeplab networks, then upsample the different scales of features into the same size of input images, finally concatenate them to produce the hierarchical visual features.

In computer vision, strong features are critical to promoting the performance [6–8]. Recent researches show that the structure of good intrinsic features is hierarchical [28, 47], which means that the features are extracted layer by layer. Moreover, CNNs with an architecture of multiple layers are able to simulate human eyes to extract the different abstract levels of features of objects, which are suitable for learning such hierarchical features. Therefore, CNNs are used to learn the hierarchical visual features from RGB images in the feature learning layer, and the operational principle is illustrated in Fig 2.

CNNs are trained layer by layer. The input and output of each layer are sets of arrays called feature maps. For each layer, the output feature maps are treated as the further abstraction of the input feature maps. Therefore, a typical CNN with *L* layers can be described as a sequence of convolution (*conv* function), rectification (*sigmoid*, *tanh* or *relu* function) and pooling (*pool* function), and utilizes softmax function to perform the final classification. It takes RGB images as input *I*, and the output convolutional features from its *l*-th layer can be formulated as $F_{l}^{\text{HVFs}}$:

$$\begin{array}{c} F_{l}^{\text{HVFs}}=pool\left[relu\left(conv\left(F_{l-1}^{\text{HVFs}}\right)\right)\right],l\in \left\{1,2,\ldots ,L\right\}, \end{array}$$ (1)

where $F_{0}^{\text{HVFs}}$ stands for input RGB images *I*.

Once the output features of all layers are generated, we upsample them into the same size of input images, then concatenate them to produce a three dimensional array called hierarchical visual features (HVFs). The HVFs can be formulated as *F*^HVFs^:

$$\begin{array}{c} \begin{array}{c} F^{\text{HVFs}}=\left[up\left(F_{1}^{\text{HVFs}}\right),\ldots ,up\left(F_{l}^{\text{HVFs}}\right),\ldots ,up\left(F_{L}^{\text{HVFs}}\right)\right]\in R^{w\times h\times n},\\ up\left(F_{l}^{\text{HVFs}}\right)\in R^{w\times h\times n_{l}}, \end{array} \end{array}$$ (2)

where *up*(⋅) stands for an upsampling function like the bilinear interpolation [12, 25], *w* and *h* are the width and height of input images *I*, *n* is the number of feature maps of the HVFs, *n*_*l*_ is the number of feature maps of the convolutional features from the *l*-th layer. For a pixel at the position (*i*, *j*), its HVFs can be denoted as $f_{i,j}^{\text{HVFs}}$:

$$\begin{array}{c} f_{i,j}^{\text{HVFs}}=F^{\text{HVFs}}\left(i,j\right)\in R^{n},1\leq i\leq w,1\leq j\leq h. \end{array}$$ (3)

The HVFs not only contain the hierarchical visual appearance information of objects, but also comprise of the implicit local contextual information among objects, which are able to improve the accuracy of scene parsing by their stronger representations. In practice, we just select the output convolutional features from several layers to produce the HVFs in order to balance the performance and computational efficiency. Through this way, not only the redundancy of feature information can be avoided, but also the computational efficiency can be improved.

#### 2.1.2 Structural learning layer

**Fig 3:**
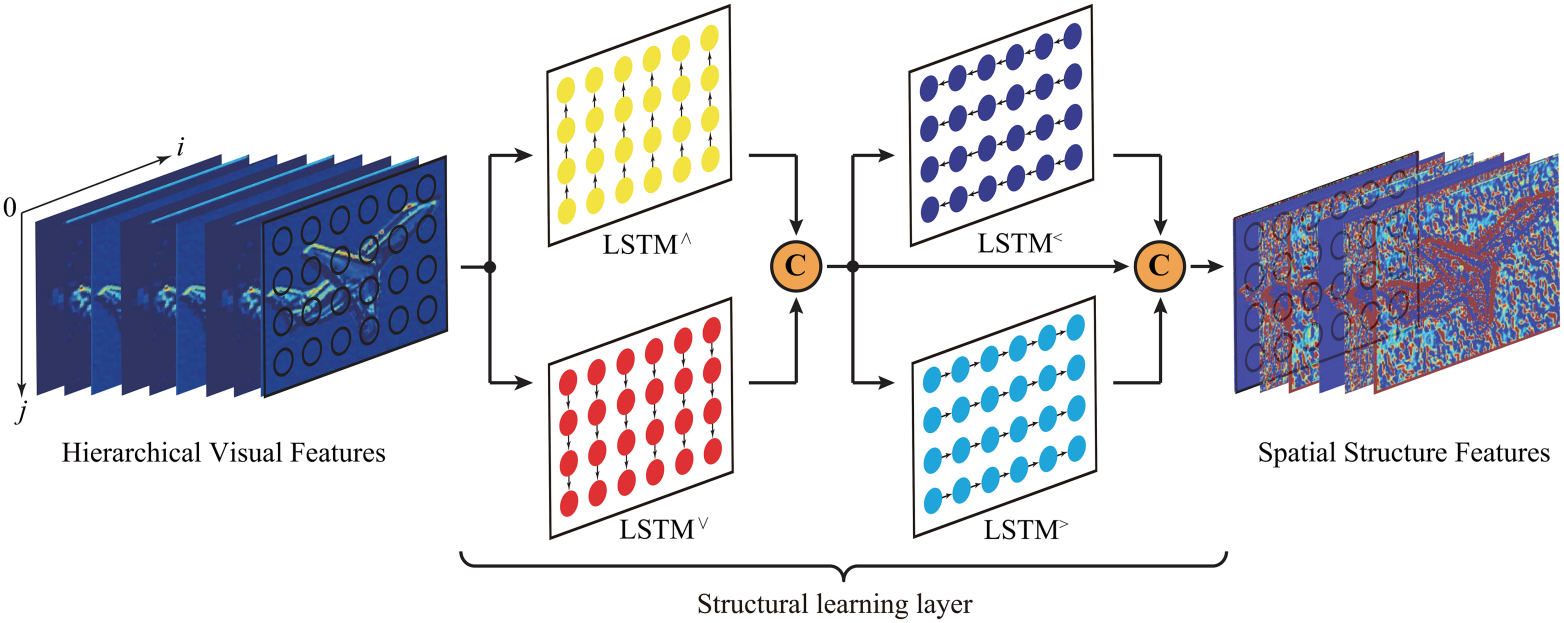
The illustration of the structural learning layer. The four uni-dimensional LSTMs sweep over the hierarchical visual features or the obtained hidden states pixel by pixel along four different directions, respectively: (1) from top to bottom (*∨*), (2) from bottom to top (*∧*), (3) from left to right (*>*), and (4) from right to left (*<*). And all the pixel-level hidden states learnt from the four different LSTMs are collected to produce the spatial structure features.

Although CNNs are able to learn the hierarchical visual features containing the precise visual appearance information of objects and the implicit local contextual information among objects, the HVFs might lead to the misclassification due to lack of the global contextual information which is used to encode the spatial dependencies among objects [10, 21, 33, 35, 37]. Moreover, long short-term memory networks with gate and memory structures [30] are close to the mechanism of human brain memorizing and forgetting information, which provide a powerful tool to infer such global contextual information by sequentially running the LSTMs over all pixels of the visual features of input images [31–38]. To remedy the drawbacks that CNNs lack the ability of inferring the global contextual information explicitly by their restricted receptive fields, we introduce the LSTMs to explicitly learn the spatial structure features which capture a global scene layout of input images, and the structural learning layer is shown in Fig 3.

For this reason, at the end of the feature learning layer we design a structural learning layer which is composed of four uni-dimensional LSTMs [32]. The four LSTMs sweep over the hierarchical visual features or the obtained hidden states pixel by pixel along four different directions, respectively: (1) from top to bottom (*∨*), (2) from bottom to top (*∧*), (3) from left to right (*>*), and (4) from right to left (*<*). The procedure of the structural learning layer can be formulated as:

$$\begin{array}{c} \begin{array}{ccc} h_{i,j}^{\text{TB}} & ={\text{LSTM}}^{\vee }\left(h_{i,j-1}^{\text{TB}},f_{i,j}^{\text{HVFs}}\right)\in R^{d},\; & \text{for}\;j=1,\ldots ,h,\\ h_{i,j}^{\text{BT}} & ={\text{LSTM}}^{\wedge }\left(h_{i,j+1}^{\text{BT}},f_{i,j}^{\text{HVFs}}\right)\in R^{d},\; & \text{for}\;j=h,\ldots ,1,\\ h_{i,j}^{\text{LR}} & ={\text{LSTM}}^{>}\left(h_{i-1,j}^{\text{LR}},\left[h_{i,j}^{\text{TB}},h_{i,j}^{\text{BT}}\right]\right)\in R^{d},\; & \text{for}\;i=1,\ldots ,w,\\ h_{i,j}^{\text{RL}} & ={\text{LSTM}}^{<}\left(h_{i+1,j}^{\text{RL}},\left[h_{i,j}^{\text{TB}},h_{i,j}^{\text{BT}}\right]\right)\in R^{d},\; & \text{for}\;i=w,\ldots ,1, \end{array} \end{array}$$ (4)

where $f_{i,j}^{\text{HVFs}}$ stands for the HVFs of a pixel at the position (*i*, *j*), $h_{i,j}^{\text{TB}}$, $h_{i,j}^{\text{BT}}$, $h_{i,j}^{\text{LR}}$, and $h_{i,j}^{\text{RL}}$ stand for the hidden states learnt from the four different LSTMs, respectively.

According to [36], for the LSTM sweeping over the HVFs from top to bottom, at the position (*i*, *j*) it takes the hidden states $h_{i,j-1}^{\text{TB}}$ and the pixel’s HVFs $f_{i,j}^{\text{HVFs}}$ as input, and its output hidden states $h_{i,j}^{\text{TB}}$ can be calculated as follows:

$$\begin{array}{c} \begin{array}{cc} gate_{f} & =\delta (W_{ff}f_{i,j}^{\text{HVFs}}+W_{fh}h_{i,j-1}^{\text{TB}}+b_{f}),\\ gate_{i} & =\delta (W_{if}f_{i,j}^{\text{HVFs}}+W_{ih}h_{i,j-1}^{\text{TB}}+b_{i}),\\ gate_{c} & =tanh(W_{cf}f_{i,j}^{\text{HVFs}}+W_{ch}h_{i,j-1}^{\text{TB}}+b_{c}),\\ c_{i,j} & =gate_{f}⊙c_{i,j-1}+gate_{i}⊙gate_{c},\\ gate_{o} & =\delta (W_{of}f_{i,j}^{\text{HVFs}}+W_{oh}h_{i,j-1}^{\text{TB}}+b_{o}),\\ h_{i,j}^{\text{TB}} & =tanh(gate_{o}⊙c_{i,j}), \end{array} \end{array}$$ (5)

where *gate*_*f*_ is the forget gate, *gate*_*i*_ is the input gate, *gate*_*c*_ is the memory gate, *gate*_*o*_ is the output gate, *W*_*ff*_, *W*_*if*_, *W*_*cf*_ and *W*_*of*_ are the recurrent gate weight matrices of the HVFs, *W*_*fh*_, *W*_*ih*_, *W*_*ch*_ and *W*_*oh*_ are the recurrent gate weight matrices of the hidden states, *δ* stands for the sigmoid function, and ⊙ stands for the point-wise product. The operation of the other LSTMs can be defined similarly.

In the end, we collect all the pixel-level hidden states learnt from the four different LSTMs to produce a three dimensional array called spatial structure features (SSFs). The SSFs can be formulated as *F*^SSFs^:

$$\begin{array}{c} F^{\text{SSFs}}=\left\{f_{i,j}^{\text{SSFs}}\right\}=\left\{\left[h_{i,j}^{\text{TB}},h_{i,j}^{\text{BT}},h_{i,j}^{\text{LR}},h_{i,j}^{\text{RL}}\right]\right\}\in R^{w\times h\times 4d}, \end{array}$$ (6)

where 4*d* is the number of feature maps of the SSFs.

The SSFs are composed of the global contextual information of objects in four different directions, respectively: (1) up, (2) down, (3) left, and (4) right, which are able to describe the spatial distributions of objects in a more comprehensive and accurate way.

#### 2.1.3 Feature fusion layer

After the procedures of the feature learning layer and structural learning layer, for a pixel at the position (*i*, *j*), it has two types of features: the hierarchical visual features $f_{i,j}^{\text{HVFs}}\in R^{n}$, which contain the precise visual appearance information, and the spatial structure features $f_{i,j}^{\text{SSFs}}\in R^{4d}$, which contain explicit global contextual information. In order to further improve the accuracy of scene parsing, we set up a feature fusion layer to achieve the multi-modal features fusion, and explore the comprehensive non-linear relationships between the HVFs and SSFs.

**Fig 4:**
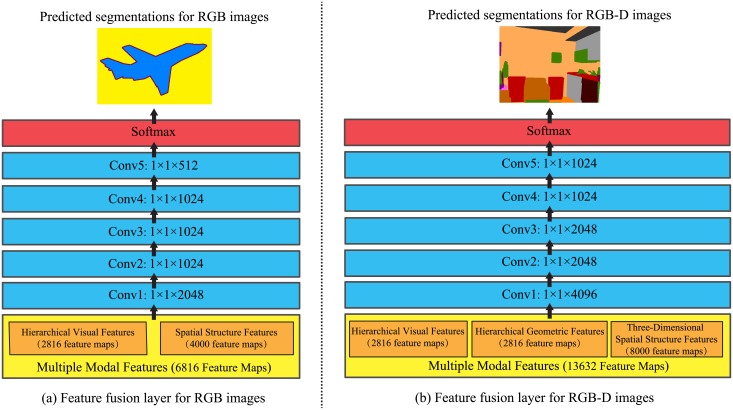
The illustration of the feature fusion layer. The hierarchical features and (three-dimensional) spatial structure features learnt from RGB-(D) images are concatenated to generate the multiple modal features (MMFs), then multiple convolutional layers are utilized to fuse the MMFs layer by layer, finally softmax function is exploited to perform pixel-wise scene labeling according to the fused MMFs.

As is illustrated in Fig 4, we concatenate the HVFs and SSFs to produce a three dimensional array called multiple modal features (MMFs), and the MMFs can be denoted as *F*^MMFs^:

$$\begin{array}{c} F^{\text{MMFs}}=\left\{f_{i,j}^{\text{MMFs}}\right\}=\left\{\left[f_{i,j}^{\text{HVFs}},f_{i,j}^{\text{SSFs}}\right]\right\}\in R^{w\times h\times \left(n+4d\right)}, \end{array}$$ (7)

then, we utilize the cascaded multiple convolutional layers (1 × 1 kernels) to fuse the MMFs layer by layer, and exploit the softmax function to perform pixel-wise scene labeling according to the fused MMFs.

The fused MMFs not only contain the visual appearance information, but also comprise of the global contextual information in four different directions, which are able to represent the comprehensive semantic information of objects. Through this way, not only the visual appearance information is utilized to perform classification, but also the global contextual information is exploited to optimize classification and avoid misclassification.

#### 2.1.4 Generator for RGB-D scene parsing

**Fig 5:**
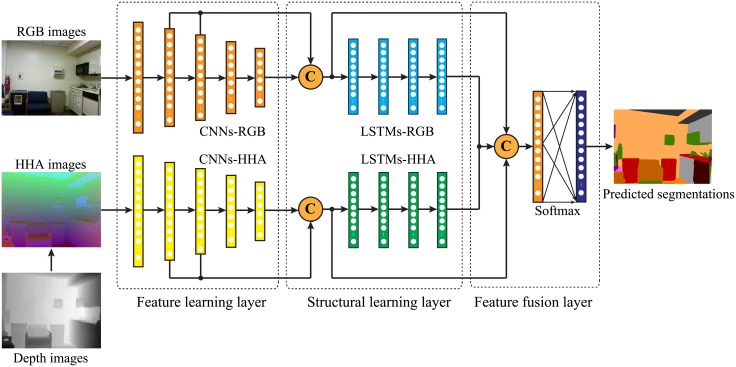
The architecture of the generator for RGB-D scene parsing. In the feature learning layer, two parallel CNNs are utilized to extract the HVFs and HGFs from RGB and HHA images, respectively. In the structural learning layer, two parallel modules composed of four uni-dimensional LSTMs sweep over the HVFs and HGFs pixel by pixel along four different directions, respectively. In the feature fusion layer, multiple convolutional layers achieve the multi-modal features fusion by learning the HVFs, HGFs, and 3D-SSFs together, then softmax function is exploited to perform pixel-wise scene labeling according to the fused MMFs.

In order to achieve scene parsing on RGB-D images, we extend the generator to make full use of depth images, and the architecture of the generator for RGB-D scene parsing is shown in Fig 5.

In the feature learning layer, another CNN is added to this layer in order to extract the hierarchical geometric features (HGFs) from HHA images which encode depth images with three channels (horizontal disparity, height above ground, and the angle the pixel’s local surface normal makes with the inferred gravity direction) at each pixel [18]. It takes HHA images as input *I*, and the output HGFs can be formulated as *F*^HGFs^:

$$\begin{array}{c} \begin{array}{cc} F_{l}^{\text{HGFs}} & =pool[relu\left(conv\left(F_{l-1}^{\text{HGFs}}\right)\right)],l\in \left\{1,2,\ldots ,L\right\},\\ F^{\text{HGFs}} & =\left[up\left(F_{1}^{\text{HGFs}}\right),\ldots ,up\left(F_{l}^{\text{HGFs}}\right),\ldots ,up\left(F_{L}^{\text{HGFs}}\right)\right]\in R^{w\times h\times n}, \end{array} \end{array}$$ (8)

where $F_{0}^{\text{HGFs}}$ stands for input HHA images *I*, *w*, *h*, and *n* are the width, height, and number of feature maps of the HGFs.

In the structural learning layer, another four uni-dimensional LSTMs are integrated into this layer so as to sweep over the hierarchical geometric features or the obtained hidden states pixel by pixel along four different directions, and this procedure can be formulated as:

$$\begin{array}{c} \begin{array}{ccc} {\bar{h}}_{i,j}^{\text{TB}} & ={\text{LSTM}}^{\vee }\left({\bar{h}}_{i,j-1}^{\text{TB}},f_{i,j}^{\text{HGFs}}\right)\in R^{d},\; & \text{for}\;j=1,\ldots ,h,\\ {\bar{h}}_{i,j}^{\text{BT}} & ={\text{LSTM}}^{\wedge }\left({\bar{h}}_{i,j+1}^{\text{BT}},f_{i,j}^{\text{HGFs}}\right)\in R^{d},\; & \text{for}\;j=h,\ldots ,1,\\ {\bar{h}}_{i,j}^{\text{LR}} & ={\text{LSTM}}^{>}\left({\bar{h}}_{i-1,j}^{\text{LR}},\left[{\bar{h}}_{i,j}^{\text{TB}},{\bar{h}}_{i,j}^{\text{BT}}\right]\right)\in R^{d},\; & \text{for}\;i=1,\ldots ,w,\\ {\bar{h}}_{i,j}^{\text{RL}} & ={\text{LSTM}}^{<}\left({\bar{h}}_{i+1,j}^{\text{RL}},\left[{\bar{h}}_{i,j}^{\text{TB}},{\bar{h}}_{i,j}^{\text{BT}}\right]\right)\in R^{d},\; & \text{for}\;i=w,\ldots ,1, \end{array} \end{array}$$ (9)

where $f_{i,j}^{\text{HGFs}}$ stands for the pixel’s HGFs at the position (*i*, *j*), ${\bar{h}}_{i,j}^{\text{TB}}$, ${\bar{h}}_{i,j}^{\text{BT}}$, ${\bar{h}}_{i,j}^{\text{LR}}$, and ${\bar{h}}_{i,j}^{\text{RL}}$ stand for the hidden states learnt from the four different LSTMs, respectively.

Once the global contextual information are learnt from the HVFs and HGFs respectively, we concatenate them to produce a three dimensional array called three-dimensional spatial structure features (3D-SSFs), and the 3D-SSFs can be formulated as *F*^3D−SSFs^:

$$\begin{array}{c} F^{3D-\text{SSFs}}=\left\{f_{i,j}^{3D-\text{SSFs}}\right\}=\left\{\left[\left[h_{i,j}^{\text{TB}},{\bar{h}}_{i,j}^{\text{TB}}\right],\left[h_{i,j}^{\text{BT}},{\bar{h}}_{i,j}^{\text{BT}}\right],\left[h_{i,j}^{\text{LR}},{\bar{h}}_{i,j}^{\text{LR}}\right],\left[h_{i,j}^{\text{RL}},{\bar{h}}_{i,j}^{\text{RL}}\right]\right]\right\}\in R^{w\times h\times 8d}, \end{array}$$ (10)

where $h_{i,j}^{\text{TB}}$, $h_{i,j}^{\text{BT}}$, $h_{i,j}^{\text{LR}}$, and $h_{i,j}^{\text{RL}}$ stand for the global contextual visual information of objects in the up, down, left, and right direction respectively, ${\bar{h}}_{i,j}^{\text{TB}}$, ${\bar{h}}_{i,j}^{\text{BT}}$, ${\bar{h}}_{i,j}^{\text{LR}}$, and ${\bar{h}}_{i,j}^{\text{RL}}$ stand for the global contextual depth information of objects in the up, down, left, and right direction respectively, and 8*d* is the number of feature maps of the 3D-SSFs.

The 3D-SSFs are composed of the global contextual visual and depth information of objects in four different directions respectively, which are able to describe the three-dimensional spatial distributions of objects in a more comprehensive and accurate way.

In the feature fusion layer, as is shown in Fig 4, we achieve the multi-modal features fusion by utilizing multiple convolutional layers to fuse the HVFs, HGFs, and 3D-SSFs together, then exploit softmax function to perform pixel-wise scene labeling according to the fused MMFs.

### 2.2 Discriminator

To make full use of each layer in the generator (scene parsing network) and obtain higher-order consistencies between the predicted segmentations and corresponding ground truths, we design a discriminator to compete with the generator via an adversarial training method.

**Fig 6:**
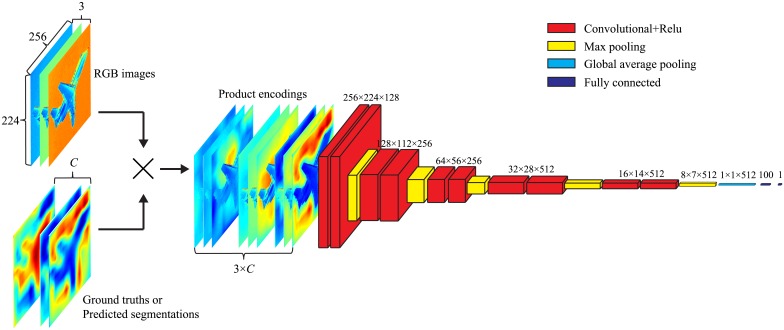
The illustration of the adversarial training layer. The discriminator takes RGB images and their corresponding ground truths (or predicted segmentations) as input, and outputs the product encodings into a convolutional neural network which consists of five stacked convolutional and max-pooling layers, a global average pooling layer, and two fully-connected layers.

As is shown in Fig 6, the architecture of the discriminator is formulated as a CNN like [41–43], which consists of five stacked convolutional and max-pooling layers, a global average pooling layer, and two fully-connected layers. The convolutional layers have a kernel size of 3 × 3, a stride of 1, a pad of 1 and use relu activations. The max-pooling layers have a kernel size of 2 × 2. The global average pooling layer takes the average of each feature map learnt from the last max-pooling layer, and outputs a one-dimensional feature vector. The first fully-connected layer utilizes tanh activations, while the second fully-connected layer employs a sigmoid activation.

In practice, we do not directly use the ground truths or predicted segmentations as the input for the discriminator, but multiply their every class probability map with the corresponding input RGB images to produce product encodings which have 3 × *C* number of feature maps and are more suitable for the discriminator to distinguish the predicted segmentations from the ground truths [41]. The procedure of the product encoding is illustrated in Fig 6. After the procedure of adversarial training, we only utilize the generator as our scene parsing network to perform scene labeling.

### 2.3 Adversarial training

Different from the standard training method [11], we optimize the generator along with the discriminator via an adversarial training method. Through this way, the generator and discriminator are like two competitors, the discriminator makes every effort to distinguish the predicted segmentations from the ground truths, whereas the generator tries its best to generate the segmentations that can cheat the discriminator [41]. That is, they play a minmax game with each other as follows [39, 40]:

$$\begin{array}{c} \begin{array}{cc} \underset{\theta_{G}}{\text{min}}\;\underset{\theta_{D}}{\text{max}}V\left(D,G\right)= & E_{T^{\left(n\right)}∼P_{T}\left(T^{\left(n\right)}\right)}\left[log\left(D\left(T^{\left(n\right)}\right)\right)\right]+\\ & E_{I^{\left(n\right)}∼P_{I}\left(I^{\left(n\right)}\right)}\left[log\left(1-D\left(G\left(I^{\left(n\right)}\right)\right)\right)\right], \end{array} \end{array}$$ (11)

where *I* = {*I*^(1)^, *I*^(2)^, …, *I*^(*N*)^} is a set of input images, *T* = {*T*^(1)^, *T*^(2)^, …, *T*^(*N*)^} is a set of corresponding ground truths, *V* stands for the objective function of the minmax game, E stands for the empirical estimate of expected value of the probability, *D*(⋅) stands for the discriminator with the architecture of CNNs, which performs the binary classification to predict whether its input is a ground truth or a predicted segmentation, *θ*_*D*_ stands for the parameters of *D*, *G*(⋅) stands for the generator like our scene parsing network, which is used to predict the right class label at every pixel of input images, and *θ*_*G*_ stands for the parameters of *G*.

#### 2.3.1 Discriminator training

Given a dataset of *N* training images, to enhance the ability of the discriminator to distinguish the predicted segmentations from the ground truths, the discriminator is trained by minimizing the following binary classification loss function *L*_*D*_:

$$\begin{array}{c} \begin{array}{cc} & L_{D}=\underset{\theta_{D}}{\text{min}}\sum_{n=1}^{N}\left[L_{BCE}\left(D\left(T^{\left(n\right)}\right),1\right)+L_{BCE}\left(D\left(G\left(I^{\left(n\right)}\right)\right),0\right)\right],D\left(\cdot \right)\in \left[0,1\right],\\ & L_{BCE}\left(x,b\right)=-\left[b\times log\left(x\right)+\left(1-b\right)\times log\left(1-x\right)\right], \end{array} \end{array}$$ (12)

where *θ*_*D*_ stands for the parameters of the discriminator *D*, *L*_*BCE*_ stands for the binary cross-entropy loss (adversarial loss) [41–43], and *D*(⋅) ∈ [0, 1] denotes the scalar probability used to predict whether its input *x* is a ground truth *T*^(*n*)^ or a segmentation prediction *G*(*I*^(*n*)^).

#### 2.3.2 Generator training

At the same time, to encourage the generator to produce the segmentations that are hard to distinguish from the ground truths by the discriminator, the generator is trained by minimizing a hybrid loss function *L*_*G*_ which encourages the generator to predict the right class label at each pixel of input images by minimizing the multi-class cross-entropy loss *L*_*MCE*_ [11, 41], meanwhile degrads the performance of the discriminator by minimizing the binary cross-entropy loss *L*_*BCE*_. Thereby, the hybrid loss function *L*_*G*_ can be formulated as follows:

$$\begin{array}{c} \begin{array}{cc} & L_{G}=\underset{\theta_{G}}{\text{min}}\sum_{n=1}^{N}\left[L_{MCE}\left(G\left(I^{\left(n\right)}\right),T^{\left(n\right)}\right)+\lambda L_{BCE}\left(D\left(G\left(I^{\left(n\right)}\right)\right),1\right)\right],\\ & L_{MCE}\left(x,y\right)=-\sum_{i=1}^{w\times h}\sum_{c=1}^{C}y_{c,i}\times log\left[x_{c,i}\right], \end{array} \end{array}$$ (13)

where *θ*_*G*_ stands for the parameters of the generator *G*, *L*_*G*_ is a linear combination of the multi-class cross-entropy loss *L*_*MCE*_ and the binary cross-entropy loss *L*_*BCE*_, and λ is the coefficient between above two loss functions.

During the procedure of the adversarial training, we update the parameters *θ*_*D*_ of the discriminator and the parameters *θ*_*G*_ of the generator in an alternating scheme [39–43]. Therefore, the training of the SIEANs proceeds by iterating between two-steps: in the first step, we fix the parameters *θ*_*G*_ of the generator *G*, and update the parameters *θ*_*D*_ of the discriminator *D* to distinguish the predicted segmentations from the ground truths, in the second step, we fix the parameters *θ*_*D*_ of the discriminator *D*, and update the parameters *θ*_*G*_ of the generator *G* to produce the segmentations that are hard to distinguish from the ground truths by the discriminator.

By this way, not only the mismatches between the predicted segmentations and corresponding ground truths in the higher-order label statistics can be detected and eliminated, but also the advantages of each layer of the generator can be exploited fully by fine-tuning its parameters with the competitive discriminator.

## 3 Experiments

### 3.1 Experimental datasets and evaluation metrics

We train our SIEANs on several standard scene parsing datasets: PASCAL VOC 2012 [48], SIFT FLOW [49], PASCAL Person-Part [50], Cityscapes [51], Stanford Background [52], NYUDv2 [53], and SUN-RGBD [54]. And the performance of the SIEANs is measured by the pixel accuracy, mean accuracy, and mean intersection over union (IoU) [11]. Let *n*_*ij*_ be the number of pixels whose true class is *i* and predicted class is *j*, *n*_*c*_ be the number of classes, then the total number of pixels whose true class is *i* can be formulated as *t*_*i*_ = ∑_*j*_ *n*_*ij*_, and above three evaluation metrics can be denoted as follows:

$$\begin{array}{c} \begin{array}{cc} & \text{Pixel}\;\text{accuracy}:\;\sum_{i=1}^{n_{c}}n_{ii}/\sum_{i=1}^{n_{c}}t_{i},\\ & \text{Mean}\;\text{accuracy}:\;\left(1/n_{c}\right)\sum_{i=1}^{n_{c}}n_{ii}/t_{i},\\ & \text{Mean}\;\text{IoU}:\;\left(1/n_{c}\right)\sum_{i=1}^{n_{c}}n_{ii}/\left(t_{i}+\sum_{j=1}^{n_{c}}n_{ji}-n_{ii}\right). \end{array} \end{array}$$ (14)

### 3.2 Experimental settings

In our experiments, we first optimize the generator (scene parsing network) via the standard training method [11], then fine-tune it via the adversarial training method [39–43]. The SIEANs is implemented on the public code framework Caffe [55], and trained on a single NVIDIA GeForce GTX TITAN X GPU with 12 GB memory.

**Fig 7:**
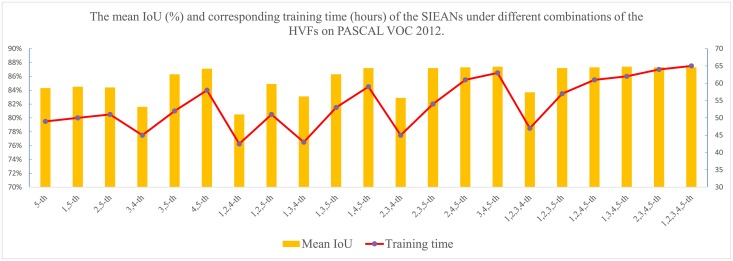
The relationship between the performance and computational efficiency of the SIEANs under different combinations of the HVFs on PASCAL VOC 2012 dataset. The number of feature maps learnt from the 1-th, 2-th, 3-th, 4-th, and 5-th layer of the Deeplab networks is 64, 256, 512, 1024, and 2048, respectively.

In the first training phase without using the adversarial loss function, we train the generator only with the multi-class cross-entropy loss function in an end-to-end fashion. In the feature learning layer, we utilize the modified Deeplab networks [12, 25] to extract the hierarchical visual features from RGB images and the hierarchical geometric features from depth images for the reason that the Deeplab networks is able to extract the dense high-resolution features by the atrous convolutions (dilated convolutions) [9, 25]. All the weights of the modified Deeplab networks are initialized with the public available pre-trained model ‘ResNet-101’ [13] which is a deeper residual learning network and can gain accuracy from considerably increased depth, and the learning rate of the feature learning layer is set to 10^−4^. In Fig 7, we present the accuracy of scene parsing and corresponding training time of the SIEANs under different combinations of the HVFs on PASCAL VOC 2012 dataset, according to the comparison results we just select the convolutional features learnt from the 2-th, 3-th, and 5-th layer of the Deeplab networks in order to balance the performance and computational efficiency of our SIEANs, and the number of feature maps from each selected layer is 256, 512, and 2048, respectively. In addition, the experimental results of the SIEANs under different combinations on other datasets are similar. In the structural learning layer, four uni-dimensional LSTMs are adopted as a whole to infer the (three-dimensional) spatial structure features which comprise of the global contextual information of objects in four different directions and are used to encode the (three-dimensional) spatial dependencies among objects. We set the number of hidden memory cells in the LSTMs to 1000, so as to ensure that the number of feature maps of the (3D)-SSFs is close to the features generated by the feature learning layer and avoid that one type of features dominates the others when the multiple modal features are fed into the feature fusion layer. We randomly initialize the weights of the LSTMs with a uniform distribution over [−0.05, 0.05], and set the learning rate of the structural learning layer to 10^−3^. In the feature fusion layer, multiple convolutional layers with a kernel size of 1 × 1 are set up to fuse all kinds of features which are learnt from above two layers and explore the comprehensive non-linear relationships between the multi-modal features. We initialize the weights of the feature fusion layer from a zero-centered Gaussian distribution with the standard deviation as 0.05, and set the learning rate of this layer to 10^−4^. Finally, we use the stochastic gradient descent algorithm to train our scene parsing network with a batch size of 20 images, momentum of 0.9, weight decay of 5^−4^, and epoch of 100.

In the second training phase with the adversarial loss function, we train a discriminator in company with the generator in an alternating scheme. We initialize all the weights of the generator with the pre-trained model learnt from the first phase, and set its learning rate to 10^−4^. We formulate the discriminator as a convolutional neural network illustrated in Fig 6. All the weights of the discriminator are initialized using a Gaussian distribution with the standard deviation as 0.05, and its learning rate is set up to 10^−4^. With the coefficient λ set to 0.05, we use the AdaGrad optimizer to train our SIEANs with a batch size of 10 images, momentum of 0.5, weight decay of 10^−4^, and epoch of 300, and alternately update the weights of the generator and discriminator at every 200 iterations.

### 3.3 Experimental results and analysis

#### 3.3.1 PASCAL VOC 2012 dataset

We first evaluate our proposed SIEANs on the well-known PASCAL VOC 2012 dataset [48], which consists of 20 object classes and one background class. This dataset is divided into three sets: a training set, a validation set, and a testing set, which contains 1464, 1449, and 1456 images, respectively. To train the SIEANs completely, we augment the original dataset with the extra annotated VOC images provided by [56], resulting in 10582 training images. To further improve the performance, we also train the SIEANs with the extra images from COCO dataset [57]. For PASCAL VOC 2012 dataset, we measure the performance of the SIEANs by the mean IoU [11].

To analyze the function of each layer in the SIEANs, we conduct an ablation study on PASCAL VOC 2012 dataset without and with COCO dataset, respectively. The comparison results are reported in Table 1. In the table, ‘CNNs’ means the accuracy of scene parsing achieved by the feature learning layer via the standard training method, ‘CNNs+LSTMs’ means the accuracy obtained by the structural learning layer via the standard training method, ‘SIEANs_STD’ means the accuracy achieved by the SIEANs via the standard training method, and ‘SIEANs’ means the accuracy obtained by the SIEANs via the adversarial training method. From the given results, we can find that the structural learning layer is the most effective component to enhance the HVFs by incorporating the global contextual information of objects in four different directions as the accuracy of scene parsing improves from 74.5% to 79.9% (without COCO dataset) and from 76.7% to 82.1% (with COCO dataset), respectively. Moreover, the feature fusion layer plays a vital role in learning more representative hybrid features encapsulated with the precise visual appearance information and the explicit global contextual information as the accuracy improves to 82.3% and 84.9%, respectively. In addition, the adversarial training method acts as an effective tool to exploit full advantages of each layer in our scene parsing network as the accuracy improves to 84.8% and 87.2%, respectively.

**Table 1:** The ablation study of the SIEANs on PASCAL VOC 2012 dataset without and with COCO dataset.

PASCAL VOC 2012 without COCO		PASCAL VOC 2012 with COCO	
Methods	Mean IoU	Methods	Mean IoU
CNNs	74.5	CNNs	76.7
CNNs+LSTMs	79.9	CNNs+LSTMs	82.1
SIEANs_STD	82.3	SIEANs_STD	84.9
**SIEANs**	**84.8**	**SIEANs**	**87.2**

In the table, ‘CNNs’ means the accuracy of scene parsing achieved by the feature learning layer via the standard training method, ‘CNNs+LSTMs’ means the accuracy obtained by the structural learning layer via the standard training method, ‘SIEANs_STD’ means the accuracy achieved by the SIEANs via the standard training method, and ‘SIEANs’ means the accuracy obtained by the SIEANs via the adversarial training method.

We also compare the SIEANs to a number of the previous state-of-the-art methods. The related results without and with COCO dataset are shown in Table 2, respectively. For the training without COCO dataset, the SIEANs outperforms the listed methods on 16 classes out of 20, and overall achieves the best mean IoU of 84.8%. For the training with COCO dataset, the SIEANs not only outperforms on 16 classes, but also achieves a mean IoU of 87.2%, which is the best among the listed methods.

**Table 2:** The comparison experimental results of the SIEANs with the previous state-of-the-art methods on PASCAL VOC 2012 dataset without and with COCO dataset. We report the mean IoU on every object class.

Methods	aero	bike	bird	boat	bottle	bus	car	cat	chair	cow	table	dog	horse	mbike	person	plant	sheep	sofa	train	tv	Mean IoU
**PASCAL VOC 2012 without COCO**																					
FCN-8s [11]	76.8	34.2	68.9	49.4	60.3	75.3	74.7	77.6	21.4	62.5	46.8	71.8	63.9	76.5	73.9	45.2	72.4	37.4	70.9	55.1	62.2
DeepLab [12]	84.4	54.5	81.5	63.6	65.9	85.1	79.1	83.4	30.7	74.1	59.8	79.0	76.1	83.2	80.8	59.7	82.2	50.4	73.1	63.7	71.6
CRF-RNN [26]	87.5	39.0	79.7	64.2	68.3	87.6	80.8	84.4	30.4	78.2	60.4	80.5	77.8	83.1	80.6	59.5	82.8	47.8	78.3	67.1	72.0
DANs_STD [41]	87.2	38.2	84.5	62.3	69.7	88.0	82.3	86.4	34.6	80.5	60.5	81.0	86.3	83.0	82.7	53.6	83.9	54.5	79.3	63.7	73.1
DANs [41]	87.1	38.5	84.9	63.2	69.7	88.0	82.5	86.8	34.5	80.3	61.5	80.9	85.8	83.3	82.6	55.0	83.5	54.7	79.7	62.9	73.3
DPNs [22]	87.7	59.4	78.4	64.9	70.3	89.3	83.5	86.1	31.7	79.9	62.6	81.9	80.0	83.5	82.3	60.5	83.2	53.4	77.9	65.0	74.1
DSMs [29]	90.6	37.6	80.0	67.8	74.4	92.0	85.2	86.2	39.1	81.2	58.9	83.8	83.9	84.3	84.8	62.1	83.2	58.2	80.8	72.3	75.3
PSPNets [21]	91.8	71.9	**94.7**	71.2	75.8	**95.2**	89.9	**95.9**	39.3	90.7	71.7	**90.5**	94.5	88.8	89.6	72.8	89.6	64.0	85.1	76.3	82.6
**SIEANs**	**93.1**	**73.8**	93.4	**75.6**	**79.0**	92.9	**92.4**	93.7	**45.1**	**91.5**	**75.3**	89.7	**94.9**	**90.7**	**91.2**	**74.8**	**92.9**	**67.1**	**87.8**	**80.3**	**84.8**
**PASCAL VOC 2012 with COCO**																					
Dilation8 [9]	91.7	39.6	87.8	63.1	71.8	89.7	82.9	89.8	37.2	84.0	63.0	83.3	89.0	83.8	85.1	56.8	87.6	56.0	80.2	64.7	75.3
ResNets [13]	87.9	41.4	89.5	72.5	80.7	93.0	87.7	91.7	39.7	83.2	53.8	85.0	85.2	82.5	85.6	59.8	85.5	40.2	87.0	77.2	76.3
PDNs [35]	87.7	42.8	89.4	73.4	80.4	93.1	88.8	91.4	39.1	83.7	51.7	84.7	84.8	83.6	86.1	60.4	87.1	42.7	87.4	78.2	76.7
DPNs [22]	89.0	61.6	87.7	66.8	74.7	91.2	84.3	87.6	36.5	86.3	66.1	84.4	87.8	85.6	85.4	63.6	87.3	61.3	79.4	66.4	77.5
DSMs [29]	94.1	40.4	83.6	67.3	75.6	93.4	84.4	88.7	41.6	86.4	63.3	85.5	89.3	85.6	86.0	67.4	90.1	62.6	80.9	72.5	77.8
LPRR [14]	92.4	45.1	94.6	65.2	75.8	95.1	89.1	92.3	39.0	85.7	70.4	88.6	89.4	88.6	86.6	65.8	86.2	57.4	85.7	77.3	79.3
DeepLab+ [25]	92.6	60.4	91.6	63.4	76.3	95.0	88.4	92.6	32.7	88.5	67.6	89.6	92.1	87.0	87.4	63.3	88.3	60.0	86.8	74.5	79.7
RefineNets [10]	94.7	64.3	94.9	74.9	82.9	95.1	88.5	94.7	45.5	91.4	76.3	90.6	91.8	88.1	88.0	69.9	92.3	65.9	88.7	76.8	83.4
PSPNets [21]	95.8	72.7	**95.0**	78.9	84.4	94.7	92.0	**95.7**	43.1	91.0	80.3	91.3	**96.3**	92.3	90.1	71.5	**94.4**	66.9	88.8	82.0	85.4
**SIEANs**	**96.1**	**75.7**	94.8	**82.4**	**86.9**	**96.2**	**93.5**	94.8	**47.7**	**93.8**	**83.6**	**91.6**	95.9	**93.8**	**92.4**	**77.0**	94.1	**69.4**	**90.5**	**84.4**	**87.2**

In this table, ‘SIEANs’ means the accuracy achieved by the SIEANs via the adversarial training method.

**Fig 8:**
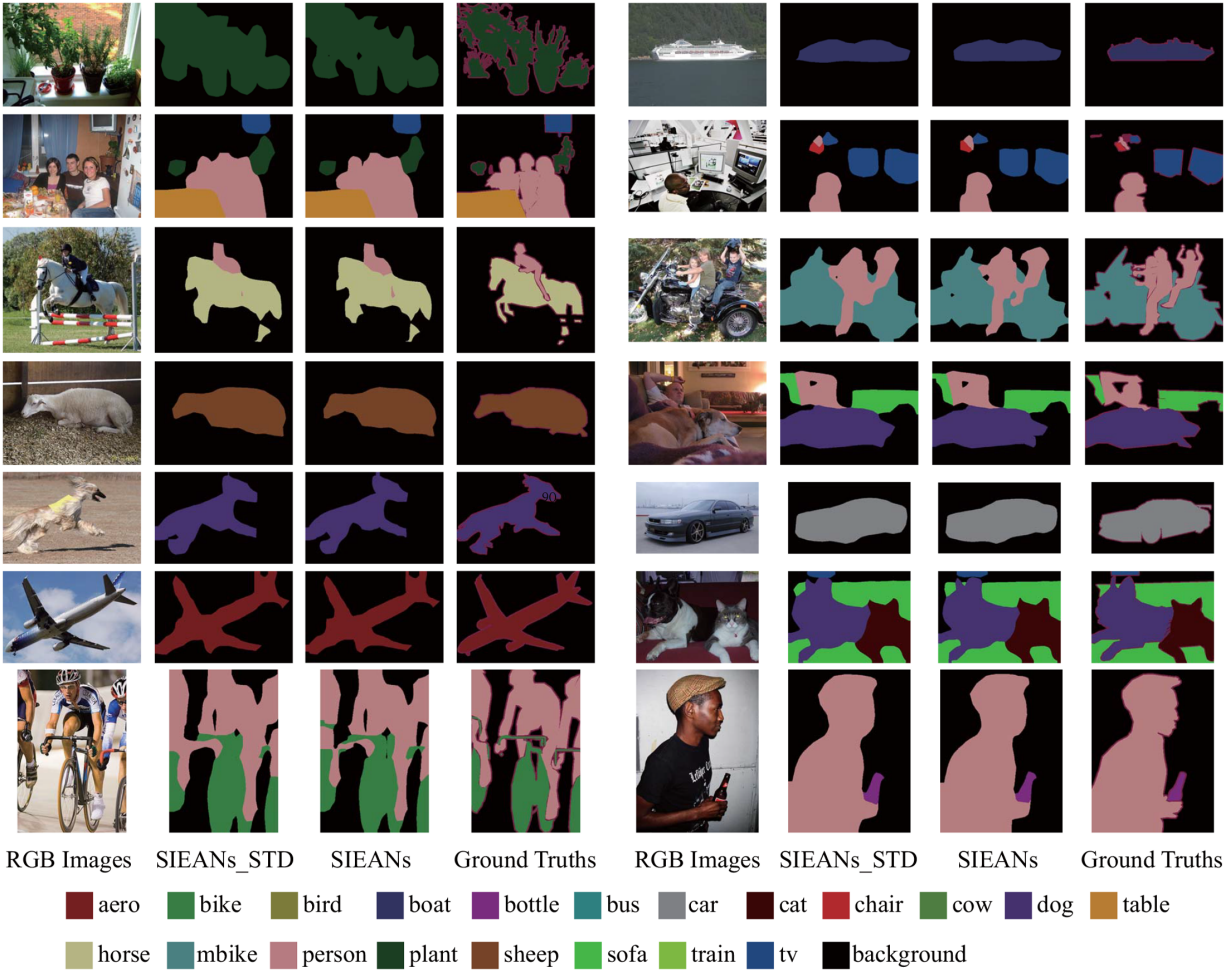
The object parsing visual results on PASCAL VOC 2012 dataset via the standard and adversarial training method. ‘SIEANs_STD’ means the visual results generated by the SIEANs via the standard training method, and ‘SIEANs’ means the visual results generated via the adversarial training method.

To further analyze the SIEANs, we present the visual results on PASCAL VOC 2012 dataset with extra COCO dataset. As can be seen in Fig 8, the SIEANs is able to produce the accurate visual results by performing pixel-wise scene labeling with the comprehensive semantic information which includes the precise visual appearance information and the explicit global contextual information. Moreover, even for the objects (‘aero’ or ‘bike’) with complex outlines, the boundaries of the predicted segmentations are also very close to the corresponding ground truths, which benefits from the explicit spatial structure inference and the adversarial training method. In addition, we also compare the visual results generated by the SIEANs via the adversarial training method to those results generated via the standard training method. It is obvious that the boundaries of the predicted segmentations generated via the adversarial training method are more close to the corresponding ground truths than those segmentations generated via the standard training method, which indicates that the adversarial training method is able to detect and correct the inconsistencies between the predicted segmentations and corresponding ground truths, and make full use of each layer in our scene parsing network to obtain higher consistencies.

To summarize, above successful results on PASCAL VOC 2012 dataset can be attributed to the following four reasons: (1) We take advantages of each layer of CNNs to efficiently extract the HVFs from RGB images, the HVFs not only contain the precise visual appearance information of objects, but also comprise of the contextual information among local areas. The more useful information we get, the more powerful the representation ability of features will be. (2) We design a novel structural learning layer, composed of four uni-dimensional LSTMs, to explicitly learn the SSFs, which contain the explicit global contextual information used to encode spatial dependencies among objects in four different directions. Based on thus SSFs, two different class pixels with the similar visual appearances can be predicted the right class labels by the difference between their additional global contextual information, meanwhile, two same class pixels with the different visual appearances can also be predicted the right labels by incorporating their similar global contextual information. (3) We set up the feature fusion layer to achieve multi-modal features fusion by learning the HVFs and SSFs together, and explore the comprehensive non-linear relationships between the multiple modal features to generate the more representative hybrid features, which encapsulate the comprehensive semantic information. Through this way, not only the visual appearance information of objects is used to estimate their classes, but also the global contextual information among objects is utilized to optimize classification and avoid misclassification. (4) We explore the adversarial training method to optimize the generator by using the adversarial loss function in addition to the standard segmentation loss function. By this way, the SIEANs not only has a higher capacity to detect and correct the mismatches in a wide range of higher-order statistics between the segmentations and ground truths, but also has a stronger ability to extract the visual appearance information from the feature learning layer, infer the global contextual information from the structural learning layer, and generate the comprehensive semantic information from the feature fusion layer.

#### 3.3.2 SIFT FLOW dataset

**Fig 9:**
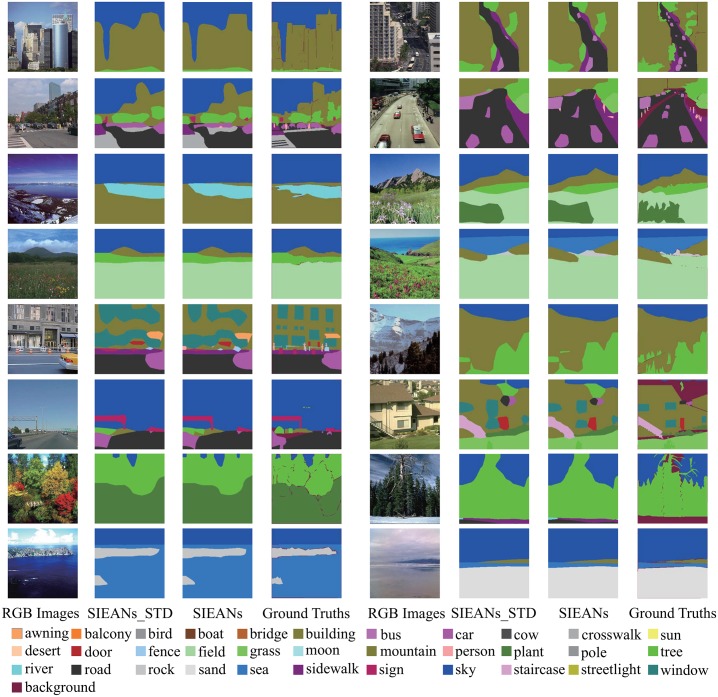
The outdoor scene parsing visual results on SIFT FLOW dataset via the standard and adversarial training method. ‘SIEANs_STD’ means the visual results generated by the SIEANs via the standard training method, and ‘SIEANs’ means the visual results generated via the adversarial training method.

We then evaluate the SIEANs on the outdoor scene parsing dataset SIFT FLOW [49]. This dataset consists of 2688 images annotated with 33 classes of semantic segmentation labels, and the images are split into 2488 training images and 200 testing images, respectively. For SIFT FLOW dataset, the performance is measured by the pixel accuracy, mean accuracy, and mean IoU, respectively [11]. The comparison experimental results are listed in Table 3 and the visual comparison results are shown in Fig 9.

**Table 3:** The comparison experimental results (pixel accuracy, mean accuracy, and mean IoU) of the SIEANs with different methods on SIFT FLOW dataset.

Methods	Pixel accuracy	Mean accuracy	Mean IoU
LHFDNs [28]	78.5	29.6	-
RCNNs [31]	77.7	29.8	-
IEDNs [47]	80.4	35.8	-
FCN-16s [11]	85.1	51.7	37.0
DAG-RNNs [59]	85.3	55.7	-
MLA-RNNs [58]	86.9	57.7	-
ELC-RNNs [37]	87.8	46.7	-
DSMs [29]	88.1	53.4	44.9
CNNs	87.5	50.3	45.2
CNNs+LSTMs	91.3	60.1	57.9
SIEANs_STD	92.0	62.5	60.7
**SIEANs**	**92.8**	**66.7**	**62.4**

In the table, ‘CNNs’ means the accuracy of scene parsing achieved by the feature learning layer via the standard training method, ‘CNNs+LSTMs’ means the accuracy obtained by the structural learning layer via the standard training method, ‘SIEANs_STD’ means the accuracy achieved by the SIEANs via the standard training method, and ‘SIEANs’ means the accuracy obtained by the SIEANs via the adversarial training method.

In Table 3, compared to the previous state-of-the-art methods, we can see that the SIEANs achieves the best performance on above three standard evaluation metrics. Furthermore, through the experimental results of the ablation study in Table 3, we can find that the accuracy of scene labeling increases higher and higher as each functional layer is integrated into the SIEANs sequentially. In addition, as can be observed in Fig 9, the visual results generated by the SIEANs via the adversarial training method have a higher consistency than those results generated via the standard training method. And above good results achieved on SIFT FLOW dataset stem from the efficient feature extraction, explicit spatial structure inference, multi-modal features fusion, and effective adversarial training method.

However, there exists a problem that it is difficult for the SIEANs to detect and recognize the small objects (‘window’, ‘car’, or ‘person’), which means that the details of small objects are lost during the training procedure. And this problem might be resulted from two points: (1) Due to the low resolution and noisy representation of small objects, the strong features are difficult to learn from their poor-quality visual appearances and structures. (2) In practice, we just select feature maps from three layers in the Deeplab networks to conduct the experiments, and some basic information of small objects might be dropped unintentionally.

#### 3.3.3 PASCAL Person-Part dataset

We also train the SIEANs to achieve object-part parsing on PASCAL Person-Part dataset [50], which provides pixel-level labels for one background and six person parts including Head, Torso, Upper/Lower Arms, and Upper/Lower Legs. This dataset is divided into sets with numbers 1716 and 1817 for training and validation, respectively. The performance of the SIEANs is measured by the mean IoU.

**Fig 10:**
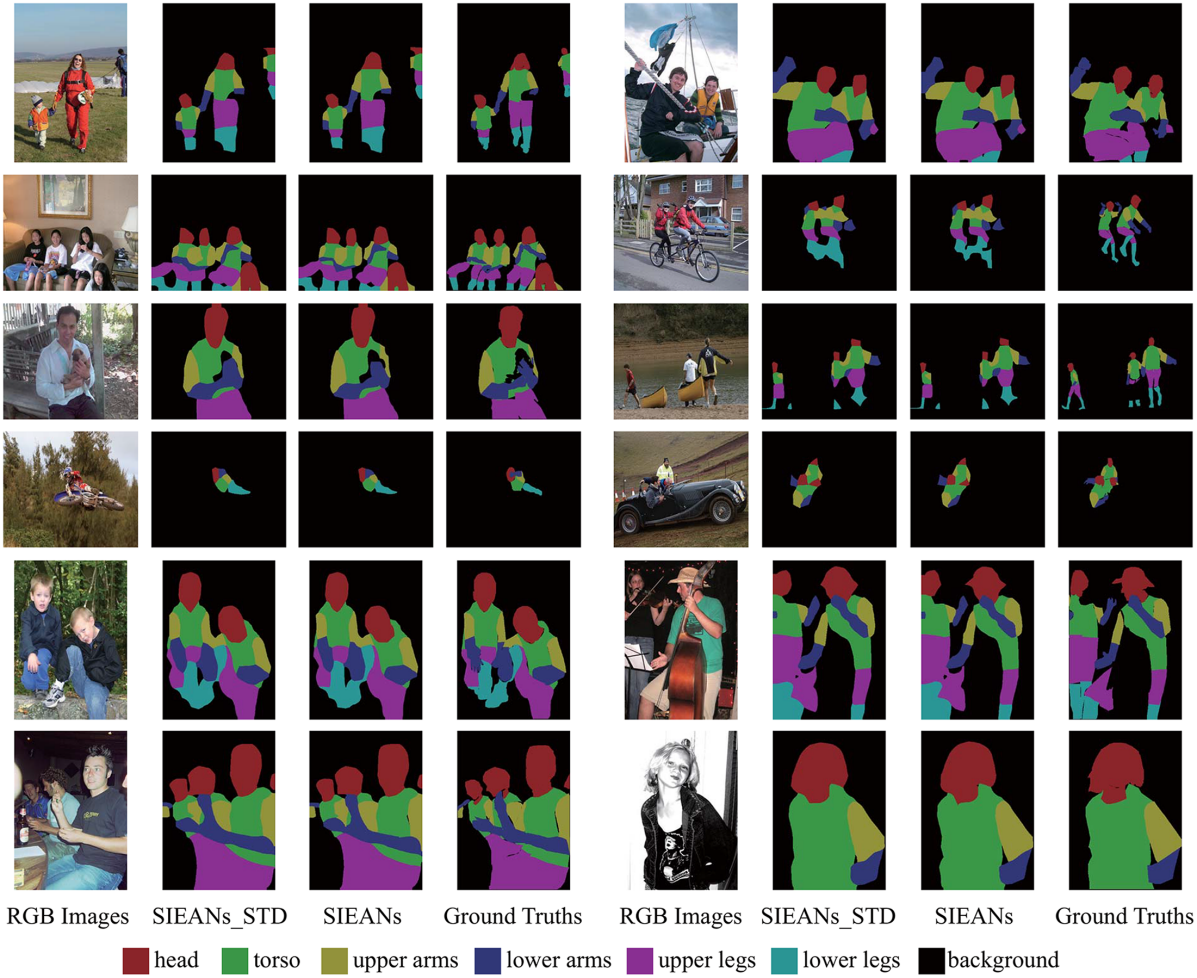
The outdoor scene parsing visual results on PASCAL Person-Part dataset via the standard and adversarial training method. ‘SIEANs_STD’ means the visual results generated by the SIEANs via the standard training method, and ‘SIEANs’ means the visual results generated via the adversarial training method.

In Table 4, we report the results of the ablation study, and we can find that the performance of the SIEANs becomes better and better as each layer is integrated into the SIEANs sequentially. Then we compare the SIEANs to the previous state-of-the-art methods in Table 5, the SIEANs achieves the best mean IoU score of 70.7. In addition, as can be seen in Fig 10, the SIEANs is able to produce the precise segmentations, and the boundaries of predicted segmentations via the adversarial training method have a higher consistency with their corresponding ground truths. And above successful results are achieved by the adversarial training SIEANs which performs classification according to the fused multiple modal features encapsulated with the precise visual appearance information and the explicit global contextual information.

**Table 4:** The ablation study of the SIEANs on PASCAL Person-Part dataset.

Methods	Mean IoU
CNNs	63.8
CNNs+LSTMs	67.1
SIEANs_STD	68.5
**SIEANs**	**70.7**

In the table, ‘CNNs’ means the accuracy of scene parsing achieved by the feature learning layer via the standard training method, ‘CNNs+LSTMs’ means the accuracy obtained by the structural learning layer via the standard training method, ‘SIEANs_STD’ means the accuracy achieved by the SIEANs via the standard training method, and ‘SIEANs’ means the accuracy obtained by the SIEANs via the adversarial training method.

**Table 5:** The comparison experimental results of the SIEANs with the previous state-of-the-art methods on PASCAL Person-Part dataset. We report the mean IoU on every object class.

Methods	head	torso	u-arms	l-arms	u-legs	l-legs	background	Mean IoU
DeepLab [12]	78.1	54.0	37.3	36.9	33.7	29.6	92.9	51.8
Attention [15]	81.5	59.1	44.2	42.5	38.3	35.6	93.7	56.4
HAZNs [16]	80.8	60.5	45.7	43.1	41.2	37.7	93.8	57.5
LG-LSTM [33]	82.7	61.0	45.4	47.8	42.3	38.0	88.6	58.0
Graph-LSTM [34]	82.7	62.7	46.9	47.7	45.7	40.9	94.6	60.2
ResNets [13]	84.2	68.8	51.2	52.2	46.8	39.4	95.3	62.6
PDNs [35]	85.6	70.0	53.3	53.2	47.5	43.7	95.6	64.1
DeepLab+ [25]	-	-	-	-	-	-	-	64.9
RefineNets [10]	-	-	-	-	-	-	-	68.6
**SIEANs**	**88.9**	**76.3**	**63.1**	**62.5**	**53.3**	**54.6**	**96.0**	**70.7**

In this table, ‘SIEANs’ means the accuracy achieved by the SIEANs via the adversarial training method.

#### 3.3.4 Cityscapes dataset

Cityscapes [51] is a very recent dataset for semantic urban scene understanding. This dataset contains 5000 high quality pixel-level annotated images with 19 classes of semantic labels, and the images are divided into sets with numbers 2975, 500, and 1525 for training, validation, and testing, respectively. For Cityscapes dataset, we train the SIEANs only with the fine data, and measure its performance by the mean IoU.

We compare the SIEANs to a number of the previous state-of-the-art methods. The related results on Cityscapes dataset are listed in Table 6. For each semantic class, the SIEANs outperforms on 12 classes out of 19, and overall achieves the best mean IoU of 80.3%. Above comparison experimental results demonstrate that the performance of the SIEANs can be improved by the explicit spatial structure inference, the multi-modal features fusion and the effective adversarial training method.

**Table 6:** The comparison experimental results of the SIEANs with the previous state-of-the-art methods on Cityscapes dataset. We report the mean IoU on every semantic class.

Methods	road	swalk	build.	wall	fence	pole	tlight	sign	veg.	terrain	sky	person	rider	car	truck	bus	train	mbike	bike	Mean IoU
CRF-RNN [26]	96.3	73.9	88.2	47.6	41.3	35.2	49.5	59.7	90.6	66.1	93.5	70.4	34.7	90.1	39.2	57.5	55.4	43.9	54.6	62.5
FCN-8s [11]	97.4	78.4	89.2	34.9	44.2	47.4	60.1	65.0	91.4	69.3	93.9	77.1	51.4	92.6	35.3	48.6	46.5	51.6	66.8	65.3
DPNs [22]	97.5	78.5	89.5	40.4	45.9	51.1	56.8	65.3	91.5	69.4	94.5	77.5	54.2	92.5	44.5	53.4	49.9	52.1	64.8	66.8
Dilation10 [9]	97.6	79.2	89.9	37.3	47.6	53.2	58.6	65.2	91.8	69.4	93.7	78.9	55.0	93.3	45.5	53.4	47.7	52.2	66.0	67.1
LPRR [14]	97.7	79.9	90.7	44.4	48.6	58.6	68.2	72.0	92.5	69.3	94.7	81.6	60.0	94.0	43.6	56.8	47.2	54.8	69.7	69.7
DeepLab+ [25]	97.9	81.3	90.3	48.8	47.4	49.6	57.9	67.3	91.9	69.4	94.2	79.8	59.8	93.7	56.5	67.5	57.5	57.7	68.8	70.4
DSMs [29]	98.0	82.6	90.6	44.0	50.7	51.1	65.0	71.7	92.0	72.0	94.1	81.5	61.1	94.3	61.1	65.1	53.8	61.6	70.6	71.6
PSPNets [21]	**98.6**	86.2	92.9	50.8	**58.8**	64.0	**75.6**	79.0	93.4	72.3	**95.4**	86.5	**71.3**	**95.9**	68.2	**79.5**	73.8	69.5	77.2	78.4
**SIEANs**	98.2	**88.5**	**94.4**	**54.7**	57.9	**69.1**	73.8	**84.3**	**94.2**	**77.5**	94.6	**89.6**	68.9	94.5	**74.0**	77.8	**79.4**	**73.4**	**79.9**	**80.3**

In this table, ‘SIEANs’ means the accuracy achieved by the SIEANs via the adversarial training method.

#### 3.3.5 Stanford Background dataset

The Stanford Background dataset [52] consists of 715 images annotated with 8 classes of outdoor scene labels. This dataset is randomly divided into two sets: a training set and a testing set, which contains 573 and 142 images, respectively. The performance of the SIEANs is measured by above three evaluation metrics.

As is listed in Table 7, compared to the previous state-of-the-art methods, our SIEANs achieves the best performance on above three evaluation metrics. In particular, compared to the DANs [41] which is a convolutional semantic segmentation network trained along with an adversarial network, we can find two trends: one trend is that the accuracy achieved by the SIEANs is obviously higher than the accuracy achieved by the DANs no matter the two networks are optimized with the adversarial loss function or not, which indicates that the structural learning layer and the feature fusion layer are able to significantly improve the performance of the SIEANs; another trend is that the accuracy achieved by above two networks via the adversarial training method are all higher than the accuracy achieved via the standard training method, which means that the adversarial training method has a strong ability to detect and correct mismatches between the predicted segmentations and corresponding ground truths, and is flexible enough to refine the parameters of the networks to get a better performance on scene parsing.

**Table 7:** The comparison experimental results (pixel accuracy, mean accuracy, and mean IoU) of the SIEANs with different methods on Stanford Background dataset.

Methods	Pixel accuracy	Mean accuracy	Mean IoU
DANs_STD [41]	73.3	66.5	51.3
DANs [41]	75.2	68.7	54.3
Zoom-out [60]	82.1	77.3	-
IL-RNNs [61]	83.1	74.8	-
MPF-RNNs [62]	86.6	79.6	-
ELC-RNNs [37]	87.8	81.1	-
CNNs	83.9	77.2	68.1
CNNs+LSTMs	88.5	82.5	77.3
SIEANs_STD	89.6	84.4	80.8
**SIEANs**	**90.9**	**87.0**	**83.4**

In the table, ‘CNNs’ means the accuracy of scene parsing achieved by the feature learning layer via the standard training method, ‘CNNs+LSTMs’ means the accuracy obtained by the structural learning layer via the standard training method, ‘SIEANs_STD’ means the accuracy achieved by the SIEANs via the standard training method, and ‘SIEANs’ means the accuracy obtained by the SIEANs via the adversarial training method.

**Fig 11:**
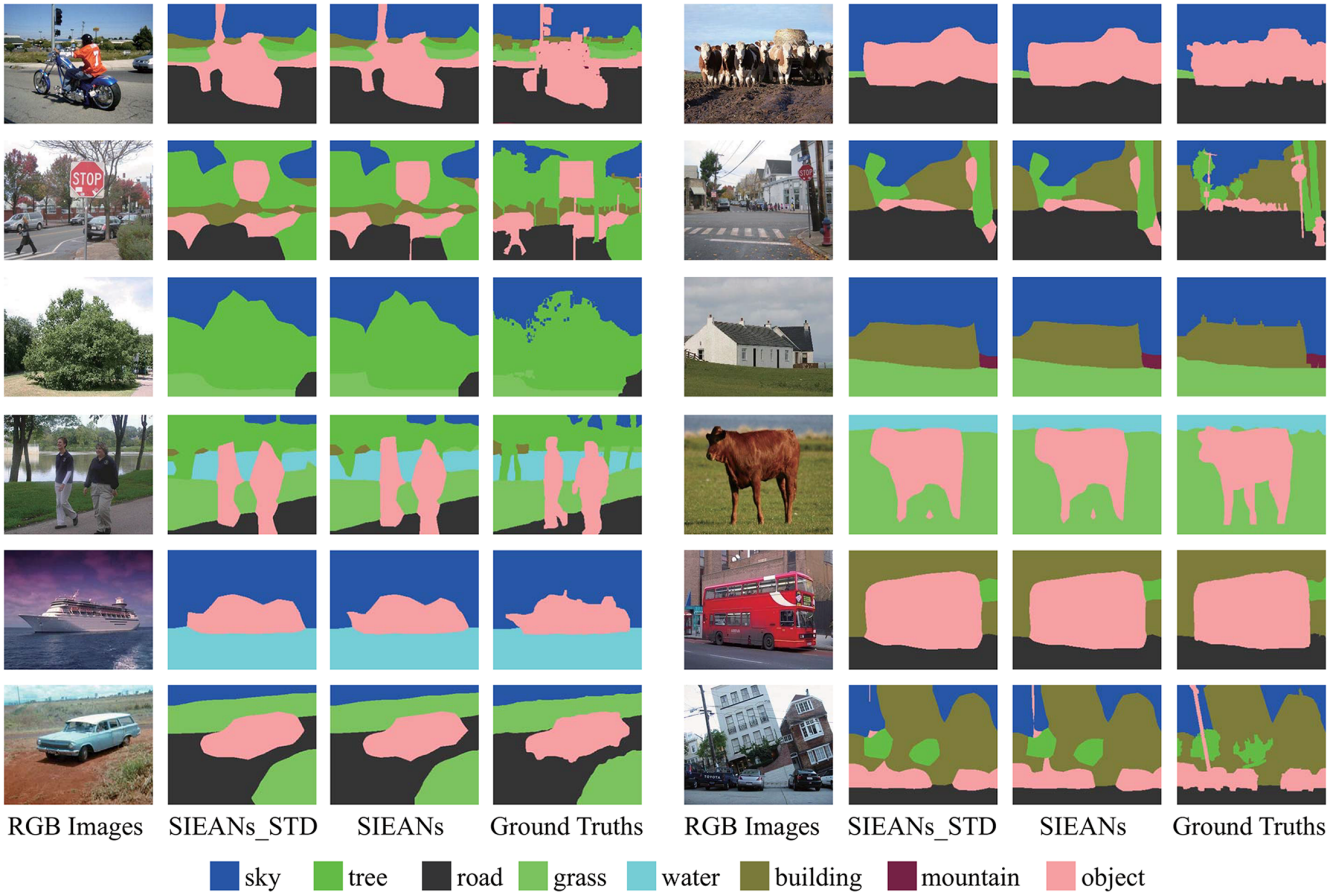
The outdoor scene parsing visual results on Stanford Background dataset via the standard and adversarial training method. ‘SIEANs_STD’ means the visual results generated by the SIEANs via the standard training method, and ‘SIEANs’ means the visual results generated via the adversarial training method.

The segmentations generated by the SIEANs via the standard and adversarial training are illustrated in Fig 11, respectively. From the given visual comparison results, we can find that the adversarial training method is able to better enforce consistencies between the predicted segmentations and corresponding ground truths, which means that the adversarial training can not only fine-tune the class probabilities over large areas, but also remove the false class labels among small areas to sharpen the boundaries of the semantic segmentations.

**Fig 12:**
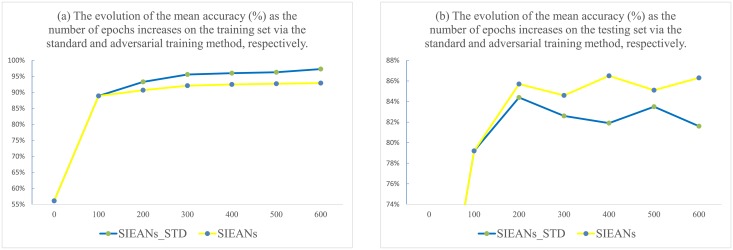
The evolution of the mean accuracy as the number of epochs increases on the training and testing set via the standard and adversarial training, respectively. ‘SIEANs_STD’ means the SIEANs optimized via the standard training method, and ‘SIEANs’ means the SIEANs optimized via the adversarial training method.

In Fig 12, we show the evolution of the mean accuracy on the training and testing set via the standard and adversarial training, respectively. As can be observed, although on the training set the accuracy achieved by the SIEANs via the standard training method is higher than the accuracy achieved via the adversarial training method as the number of epochs increases, on the testing set the accuracy achieved via the adversarial training method is higher than the accuracy achieved via the standard training method, which means that the adversarial training method is able to improve the accuracy on the testing set by reducing the overfitting and generating a regularization effect.

#### 3.3.6 NYUDv2 dataset

We further evaluate our SIEANs on the indoor scene parsing dataset NYUDv2 [53]. This dataset contains 1449 RGB-D images and provides 40 classes of semantic segmentation labels. The training set consists of 795 images and the testing set consists of 654 images. We first train the SIEANs only with RGB images, then fine-tune it with extra depth images. To make full use of depth information, we utilize the modified SIEANs to achieve scene parsing on RGB-D images, and the architecture of our SIEANs for RGB-D scene parsing is show in Fig 5. For NYUDv2 dataset, we measure the performance by the pixel accuracy, mean accuracy, and mean IoU.

**Fig 13:**
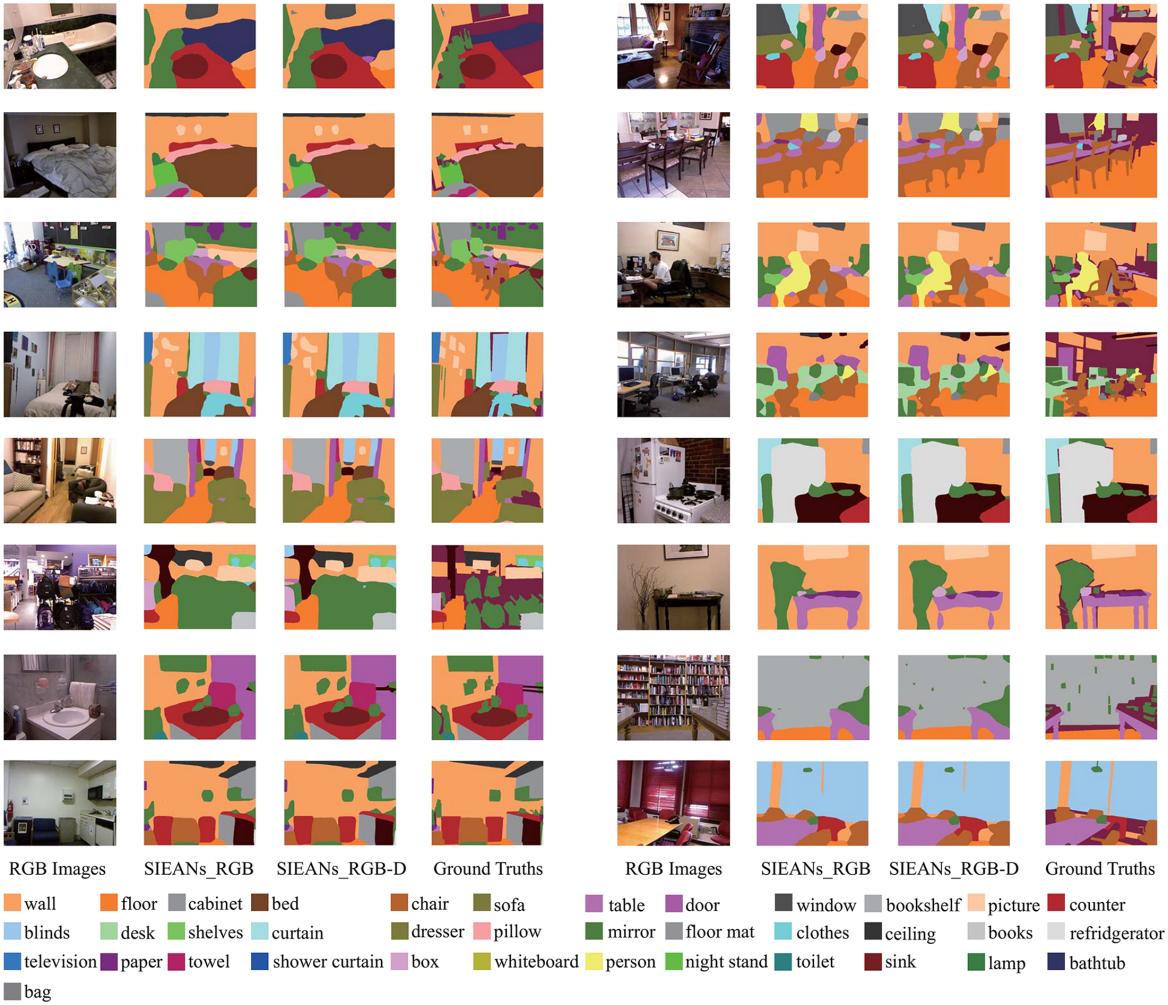
The indoor scene parsing visual results on NYUDv2 dataset including or not including depth images. ‘SIEANs_RGB’ means the visual results generated by the SIEANs on RGB images, and ‘SIEANs_RGB-D’ means the visual results generated by the SIEANs on RGB-D images.

The experimental results of the ablation study on NYUDv2 dataset including or not including depth images are listed in Table 8, respectively. From the given results, we can find two trends: one trend is that the accuracy of scene parsing improves higher and higher as the SIEANs is gradually integrated layer by layer no matter the NYUDv2 dataset includes depth images or not; another trend is that the accuracy achieved by the SIEANs with different configurations on RGB-D images are all higher than the accuracy achieved by the corresponding networks on RGB images. Moreover, we compare the SIEANs to the previous state-of-the-art methods in Table 9, and the SIEANs achieves the best performance on above three evaluation metrics. As can be seen in Fig 13, the boundaries of the segmentations generated by the SIEANs on RGB-D images are much closer to the corresponding ground truths than those segmentations generated on RGB images.

**Table 8:** The ablation study of the SIEANs on NYUDv2 dataset including or not including depth images.

NYUDv2 not including depth images				NYUDv2 including depth images			
Methods	Pixel accuracy	Mean accuracy	Mean IoU	Methods	Pixel accuracy	Mean accuracy	Mean IoU
CNNs	70.6	54.2	41.5	CNNs	72.3	56.8	44.5
CNNs+LSTMs	72.9	57.5	44.9	CNNs+LSTMs	75.7	61.4	49.7
SIEANs_STD	74.1	59.4	47.8	SIEANs_STD	76.4	62.9	51.2
**SIEANs**	**75.0**	**60.6**	**49.3**	**SIEANs**	**77.0**	**64.2**	**53.8**

In the table, ‘CNNs’ means the accuracy of scene parsing achieved by the feature learning layer via the standard training method, ‘CNNs+LSTMs’ means the accuracy obtained by the structural learning layer via the standard training method, ‘SIEANs_STD’ means the accuracy achieved by the SIEANs via the standard training method, and ‘SIEANs’ means the accuracy obtained by the SIEANs via the adversarial training method.

**Table 9:** The comparison experimental results (pixel accuracy, mean accuracy, and mean IoU) of the SIEANs with the previous state-of-the-art methods on NYUDv2 dataset including or not including depth images.

Methods	Training data	Pixel accuracy	Mean accuracy	Mean IoU
JDFLE [17]	RGB-D	51.6	29.2	-
BUPDNs [18]	RGB-D	58.3	29.6	30.7
FCN-32s [11]	RGB	60.0	42.2	29.2
IGCDNs [24]	RGB-D	50.7	43.9	-
FCN-HHA [11]	RGB-D	65.4	46.1	34.0
LSTM-CF [36]	RGB-D	-	49.4	-
DSMs [29]	RGB	70.0	53.6	40.6
RefineNets [10]	RGB	73.6	58.9	46.5
SIEANs	RGB	75.0	60.6	49.3
**SIEANs**	RGB-D	**77.0**	**64.2**	**53.8**

In this table, ‘SIEANs’ means the accuracy achieved by the SIEANs via the adversarial training method.

Based on above competitive performance on NYUDv2 dataset, we can conclude: (1) We utilize CNNs to extract the HGFs from depth images, and the HGFs contain the auxiliary geometric information which is able to enrich the visual appearances of objects in company with the HVFs. (2) We use the structural learning layer to explicitly infer the 3D-SSFs, and the 3D-SSFs comprise of the global contextual visual and depth information which is able to describe the three-dimensional spatial distributions of objects in a more comprehensive and accurate way. (3) We achieve the multi-modal features fusion by learning the HVFs, HGFs, and 3D-SSFs together, and the fused MMFs consist of the comprehensive semantic information of objects. (4) We exploit the adversarial training method to make full use of each layer of the generator (scene parsing network).

#### 3.3.7 SUN-RGBD dataset

We also evaluate the SIEANs for RGB-D scene parsing on SUN-RGBD dataset [54], which is the largest dataset currently available. This dataset consists of 10355 RGB-D images and provides 37 classes of semantic segmentation labels. The training set consists of 5285 images and the testing set consists of 5050 images. For SUN-RGBD dataset, we measure the performance of the SIEANs by above three evaluation metrics.

In Table 10, no matter these networks are trained with depth images or not, the SIEANs outperforms the previous state-of-the-art methods on the pixel accuracy, mean accuracy, and mean IoU, respectively. And above good results mainly benefit from the explicit three-dimensional spatial structure inference and the effective adversarial training method.

**Table 10:** The comparison experimental results (pixel accuracy, mean accuracy, and mean IoU) of the SIEANs with the previous state-of-the-art methods on SUN-RGBD dataset including or not including depth images.

Methods	Training data	Pixel accuracy	Mean accuracy	Mean IoU
Liu et al. [49]	RGB-D	-	10.1	-
Ren et al. [19]	RGB-D	-	36.3	-
Bayesian [20]	RGB	71.2	45.9	30.7
LSTM-CF [36]	RGB-D	-	48.1	-
DSMs [29]	RGB	78.4	53.4	42.3
RefineNets [10]	RGB	80.6	58.5	45.9
SIEANs	RGB	81.6	60.4	50.1
**SIEANs**	RGB-D	**84.8**	**63.0**	**52.7**

In this table, ‘SIEANs’ means the accuracy achieved by the SIEANs via the adversarial training method.

## 4 Conclusion

In this paper, we propose a novel adversarial training model embedded with the structural learning for pixel-wise scene labeling. The generator of our SIEANs, composed of three types of layers, provides a powerful framework to perform explict spatial structure inference. Moreover, the adversarial training method makes it possible for the generator to obtain a higher consistency between the predicted segmentations and corresponding ground-truths in company with the competitive discriminator. Furthermore, the SIEANs can be easily extended to achieve scene parsing on RGB-D images, and the accuracy of scene parsing achieved with depth information can be boosted.

Although our SIEANs is able to achieve the state-of-the-art performance in a supervised manner, our training method requires large amount of pixel-level annotated data, which is highly prohibitive to obtain. To this end, in future we will explore a semi-supervised or an unsupervised method to train our proposed SIEANs, which is more suitble in practice.
